# Endomyocardial biopsy in patients with cardiomyopathy of unknown origin: does specialized center experience apply to a tertiary care hospital?

**DOI:** 10.1186/s13104-016-2263-4

**Published:** 2016-10-10

**Authors:** Ulrich Tebbe, Karin Bramlage, Fiete John, Dirk Härtel, Ralf Felgendreher, Kathrin Machalke, Reinhard Kandolf, Peter Bramlage

**Affiliations:** 1 Clinic for Cardiology, Angiology and Internal Intensive Medicine, Herz-Kreislauf-Zentrum, Klinikum Lippe, Detmold, Germany; 2 Institute for Pharmacology and Preventive Medicine, Cloppenburg, Germany; 3 Department of Molecular Pathology, University of Tübingen, Tübingen, Germany

**Keywords:** Cardiomyopathy, Myocardial biopsy, Myocarditis, Cardiac amyloidosis, Viral

## Abstract

**Background:**

In patients with cardiomyopathy of unknown origin, endomyocardial biopsy provides the possibility of improved diagnosis and tailored treatment. Specific guidance has been developed based on cardiovascular centre of excellence experience but it is unknown if the benefits also extend into the tertiary care hospital setting.

**Methods:**

Endomyocardial biopsies was performed in patients with cardiomyopathy of unknown origin. The outcomes were mirrored against the current ESC recommendations.

**Results:**

A total of 57 patients with cardiomyopathy of unknown origin underwent endomyocardial biopsy with a mean age of 54 years and 28 % being women. In 17 patients (30 %), viruses were detected in the biopsy material, in 6 patients (11 %) cardiac amyloidosis was found of which 3 had also a positive test for viruses. The overall mortality rate was 18 % in the mean follow up period of 30 months, with a rate of 24 % in those with virus detection (mean FU 24 months) and 15 % in those without virus detection (mean FU 31 months. Death rates were 83 % in patients with cardiac amyloidosis (mean FU 10 months).

**Conclusion:**

We conclude that, limited by uncertainty stemming from the small number of included patients, endomyocardial biopsy may not prove to have a clinical impact on treatment decisions and outcomes in a tertiary care hospital setting. We consider cardiac amyloidosis to be an exception, since the mortality rate with or without concomitant virus load was extremely high.

## Background and objectives

In 31 % of patients with cardiomyopathy of unknown origin, clinical assessment of the cause is often inaccurate. However, a definitive diagnosis with high specificity can be reached for 75 % of these patients using endomyocardial biopsy [1]. Involvement of the heart muscle in infectious-inflammatory or autoimmune disease can only be detected by examining myocardial biopsy samples. Specific myocardial disorders associated with individual prognoses and treatment options are rarely diagnosed through non-invasive tests [2]. Non-invasive imaging techniques, such as cardiac magnetic resonance imaging (MRI), can identify aspects representative of certain causes. However, to confirm the diagnosis, endomyocardial biopsy is essential [3]. When cardiac amyloidosis is suspected, endomyocardial biopsy is the gold standard for achieving an accurate diagnosis [4]. Amyloidosis patients with cardiac involvement have a poor prognosis. For them, an early and specific diagnosis is critical for allowing rapid introduction of modern therapy and an increase in treatment success [5].

In a joint scientific statement from the American Heart Association (AHA), the American College of Cardiology (ACC), and the European Society of Cardiology (ESC) [6], the importance of endomyocardial biopsy in the treatment of cardiovascular diseases was clearly established (Table 1). This recommendation was developed from data usually obtained in larger, highly specialized centres for the care of patients with cardiomyopathy. We were interested to see whether these recommendations as well as the specialized centre experience also applies to the situation in a tertiary care centre, with responsibility for a large variety of patients.

**Table 1:** Patient characteristics

	Total (n = 57)	Virus-positive (n = 17)	Virus-negative (n = 40)	Amyloidosis-positive (n = 6)	Amyloidosis-negative (n = 51)
Women	16 (28 %)	7 (41 %)	9 (23 %)	1 (17 %)	15 (29 %)
Age (years)	54	49	55	73	51
Mean EF	50 %	51 %	50 %	64 %	49 %
EF <35 %	13 (23 %)	7 (41 %)	6 (15 %)	0 (0 %)	13 (26 %)
Virus-positive	17 (30 %)	17 (100 %)	0 (0 %)	3 (50 %)	14 (28 %)
Amyloidosis-positive	6 (11 %)	3 (18 %)	3 (8 %)	6 (100 %)	0 (0 %)
Death	10 (18 %)	4 (24 %)	6 (15 %)	5 (83 %)	5 (10 %)
Mean follow-up (months)	30	28	31	10	33

In the presently reported survey, endomyocardial biopsies were performed on patients with cardiomyopathy of unknown cause, and the outcomes compared with the aforementioned guidelines of the AHA, ACC, and ESC [6]. A special consideration was given to the question whether we may find proof that these guidelines are applicable for clinical decision making and patient outcomes in a tertiary care centre.

### Study design and research methods

Between 2003 and 2013, a total of 14,775 diagnostic coronary angiographies were performed in the cardiology centre of the Lippe Clinic, Detmold. From these, 1302 patients (8.8 %) were diagnosed with cardiomyopathy. Based on their history, clinical course, and differential diagnosis and in accordance with the specific guidance [6] the decision for left ventricular myocardial biopsies (minimum 3–5 biopsies at different locations) was made in 57 patients (4.4 % of those with cardiomyopathy).

Patients were anonymized upon data entry and therefore ethical approval was not required [7, 8]. We obtained written informed consent for the procedure itself and specific consent from the patient described in the case report.

### Histopathological and immunohistochemical analysis of endomyocardial biopsy samples

Examination of the samples was performed at the Institute of Molecular Pathology (Prof. Kandolf) of the University of Tübingen. Sections of the tissue samples were stained with haematoxylin, eosin, Masson’s trichrome stain, and Giemsa. Immunohistochemical studies were also performed on tissue samples fixed with 4 % neutral buffered formaldehyde solution [9]. Biopsy samples stained with Masson’s trichrome and haematoxylin-eosin (HE), and those embedded in paraffin, were examined by light microscopy. In the event of a diagnosis of cardiac amyloidosis, samples would also be histologically stained with Congo red. The histological analysis was performed according to the Dallas criteria supplemented by immunohistochemistry for the evaluation of an ongoing inflammatory reaction [10]. For immunohistochemical staining, tissues were treated with various antibodies conjugated to an avidin-biotin-peroxidase complex (Vectastain-Elite, ABC Kit, Vector, Burlingame, CA, USA).

### Molecular and virological analyses

Other biopsy samples were snap-frozen or fixed in RNAlater (Ambion Inc., Foster City, CA, USA) for detection of the virus genome by polymerase chain reaction [PCR-Nested-PCR/reverse transcription PCR (RT-PCR)]. This was carried out to detect the following viruses: Parvovirus B19, human herpes virus type 6, enteroviruses (coxsackie and echo viruses), Epstein Barr virus, influenza virus A and B, adenovirus, herpes simplex virus, and varicella zoster virus [11]. Glyceraldehyde-3-phosphate dehydrogenase was used as an internal control to indicate successful isolation of nucleic acids. A biopsy was designated as positive if viral genetic material was detected by PCR. Specificity was ensured by automatic DNA sequencing of viral amplification products [12].

### Statistics

For descriptive statistics, frequency (percentage) and mean values are reported. The probability of survival after myocardial biopsy is presented as a Kaplan Meier curve.

## Results

Among the 57 patients documented (Table 1), the proportion of women was 28 % and the mean age was 54 years. A total of 17 patients were virus positive (30 %), 6 were amyloid positive (11 %) and three were virus and amyloid positive (5 % of all, 50 % of those with cardiac amyloidosis).

**Fig. 1:**
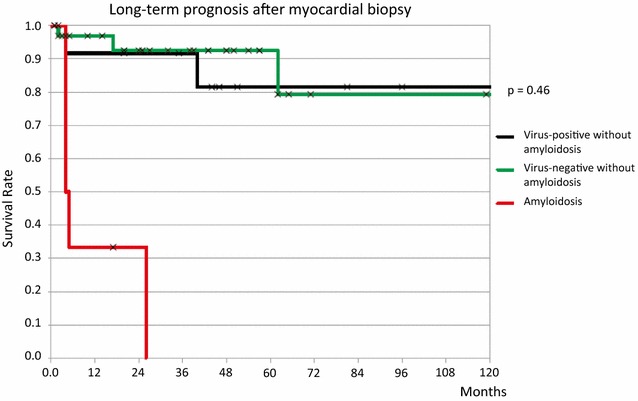
Long-term prognosis after myocardial biopsy

Virus positive patients had an average age of 49 years with a fairly good ejection fraction (mean 51 %). None of them was receiving antiviral or immunosuppressive therapy. After a mean follow-up of 28 months 4 of the virus positive patients had died (24 %) which was nominally larger than in the virus negative group (6 patients or 15 % after 31 months of follow-up). In Fig. 1, survival rate is divided into virus-positive and virus-negative patients without amyloidosis, and those with amyloidosis. The difference between the two groups without amyloidosis did not prove significant (p = 0.46).

Six patients were diagnosed with amyloidosis, with these being on average 22 years older than those without (Table 1). Three of these also tested positive for virus genome. Again, none of the patients received antiviral or immunosuppressive therapy. While the EF was rather high in these patients (64 %), the prognosis was particularly poor with 83 % (5 out of 6 patients) dying within the first 10 months. This was substantially higher than the death rate in amyloid negative patients that was 10 % after a mean follow-up of 33 months. The surviving virus-positive patient with transthyretin-related amyloidosis (ATTR) is currently undergoing treatment (see ‘‘Case report’’ section).

Biopsy sampling was carried out safely and without complications. None of the 57 patients suffered cardiac tamponade or suffered from a neurological deficit.

## Case report

A 71-year-old patient presented at a neurology clinic with increasing unsteadiness and numbness of the legs. He also complained of a sensation of pressure in the chest during exercise. Two years earlier, he had undergone an ambulatory coronary angiography in which a left bundle branch block and mildly impaired systolic function were observed, without evidence of stenotic heart disease. Owing to the presence of peripheral polyneuropathy, a biopsy was taken, and this tested positive for amyloidosis. A cardiac investigation was additionally performed because of the thoracic discomfort. Echocardiography revealed severe restrictive cardiomyopathy. Contrast-enhanced cardiac MRI showed no evidence of intramyocardial pathology attributable to amyloidosis. However, in a myocardial biopsy, amyloidosis was detected. Amyloid deposits were also detected in an Ileum biopsy. Genetic testing revealed a heterozygous status for mutation c.233T > A (p.Lau78His) in the TTR gene. Thus, the diagnosis of systemic hereditary ATTR was reached. The patient was started on Tafamidis. Since the beginning of treatment (17 months so far), no disease progression has been observed.

## Discussion

Limited by uncertainty stemming from the small number of included patients, endomyocardial biopsy may not prove to have a clinical impact on treatment decisions and outcomes in a tertiary care hospital setting. We consider cardiac amyloidosis to be an exception, since the mortality rate with or without concomitant virus load was extremely high (83 % in 10 months).

### Viral myocarditis

Viral myocarditis is an important causal factor in progression to dilated cardiomyopathy [13–16], currently the most common reason for heart transplantation [16]. Kindermann et al. studied 181 patients with suspected viral myocarditis and came to the conclusion that a higher NYHA class, immunohistological inflammatory characteristics, and a lack of β-blocker therapy were associated with a poor prognosis, irrespective of histological criteria of myocarditis (Dallas criteria) or virus genome detection [17]. Schwab et al. indicated that early diagnosis of myocarditis is crucial, as positive patient outcomes can occur only when the myocardial regenerative potential is still present. As the disease progresses, it may lead to irreversible myocardial injury, which would lead to development of heart failure and its progression [18]. There is one large study by Felker et al. [2] showing no difference in survival between cases of idiopathic and myocarditis based cardiomyopathy. They grouped patients into the categories idiopathic cardiomyopathy, peripartum cardiomyopathy and cardiomyopathy due to myocarditis, ischemic disease, infiltrative myocardial disease, hypertension, HIV- infection, connective tissue disease, substance abuse, therapy with doxorubicin and other causes. Felker et al. conclude that the underlying cause of HF has prognostic value in patients with unexplained cardiomyopathy. Survival among the patients with cardiomyopathy due to myocarditis, substance abuse, hypertension, connective-tissue, disease, or other causes did not differ significantly from that among patients with idiopathic cardiomyopathy. The ESC Working Group on Myocardial and Pericardial Diseases reported that in up to 30 % of biopsy-proven myocarditis, cardiomyopathy develops, resulting in poor prognosis [19]. Myocarditis covers a wide clinical spectrum and displays no pathognomonic symptoms [3]; therefore, it is often only detected at an advanced stage. Cooling et al. state in their review that such numerous and chronic viral infections and post-infectious autoimmune inflammations of the myocardium are treatable and, therefore, early myocardial biopsy clarification is necessary in order to prevent the emergence of irreversible therapy-refractory heart muscle damage [20]. To date, there is no evidence-based treatment recommendation, since the clinical trial situation is inadequate [3]. Immunosuppressive therapy appears to be effective in patients with virus-negative chronic myocarditis. However, this treatment option, similar to immunomodulatory and antiviral strategies, has yet to be investigated in randomised placebo-controlled trials [3].

### Cardiac amyloidosis

In this study, it became clear that detection of cardiac amyloidosis was associated with a very poor prognosis. Only one of the 6 patients diagnosed with cardiac amyloidosis survived to receive adequate therapy. Cardiac involvement usually manifests itself with nonspecific symptoms (decrease in performance, increase in exertional dyspnoea, peripheral oedema, palpitations, or syncope) [21]. For the diagnosis of cardiac amyloidosis, endomyocardial biopsy is the gold standard [4]. Through early and precise diagnosis, targeted therapy can be initiated. In our case study (patient with ATTR), this resulted in the successful start of therapy with the “orphan drug” Tafamidis.

Furthermore we had three out of six patients in which virus myocarditis was superimposed on a cardiac amyloidosis. Although previously published [22], this is a rare observation. Lim et al published a similar case report in 2006 where a 57 year-old women presented with acute myocarditis with a concomitant amyloidosis [22]. The patients showed arrhythmia due to a conduction disorder. The authors concluded that myocardial biopsies have proven helpful with regard to the diagnosis of acute myocarditis superimposed on amyloidosis, especially in patients which present conduction disorder and rather preserved ventricular function.

## Limitations

This is a small case series of patients with diagnosed cardiomyopathy undergoing left ventricular myocardial biopsy. Data were collected over a time-period of 10 years in a tertiary care hospital. They are likely to be affected by bias in patient selection for biopsy and are subject to the bias clinical routine may impose in this setting. They are not meant to challenge larger observations or trials, but they rather reflect clinical reality beyond large clinical centres specialising in cardiomyopathies. Further limitations include the lack of further data (such as immunospecific stain etc.) on the patients diagnosed with amyloidosis, the lack of a full documentation on the indication for biopsy, the presence of active myocarditis, and on concomitant medication and treatment.

## Conclusions

Patients with an indication for myocardial biopsy according to the ESC Working Group on Myocardial and Pericardial Diseases have an adequate long-term prognosis, with no difference between those having virus genome detected and those not. The observation is however limited by the small sample size a tertiary care centre is able to collect even in a 10 year frame. On the other hand the data illustrate the considerable impact of cardiac amyloidosis on outcomes. Left ventricular myocardial biopsy was not associated with major complications affecting the decision to go for biopsy. We conclude that, limited by uncertainty stemming from the small number of included patients, endomyocardial biopsy may not prove to have a clinical impact on treatment decisions and outcomes in a tertiary care hospital setting except for a clinical suspicion of amyloidosis.

## Abbreviations

ACC: American College of Cardiology
AHA: American Heart Association
ARVD/C: arrhythmogenic right ventricular dysplasia/ cardiomyopathy
ATTR: transthyretin- related amyloidosis
DCM: dilated cardiomyopathy
EF: ejection fraction
ESC: European Society of Cardiology
HCM: hypertrophic cardiomyopathy
HE: haematoxylin- eosin
MRI: magnetic resonance imaging
PCR: polymerase chain reaction
RT- PCR: reverse transcription PCR
SSA: systemic senile amyloidosis
TTR: transthyretin
TTR-FAP: transthyretin familial amyloid polyneuropathy

## References

[1] Ardehali H, Qasim A, Cappola T, Howard D, Hruban R, Hare JM, Baughman KL, Kasper EK (2004). Endomyocardial biopsy plays a role in diagnosing patients with unexplained cardiomyopathy. Am Heart J.

[2] Felker GM, Thompson RE, Hare JM, Hruban RH, Clemetson DE, Howard DL, Baughman KL, Kasper EK (2000). Underlying causes and long-term survival in patients with initially unexplained cardiomyopathy. N Engl J Med..

[3] Kindermann I, Kindermann M, Mahfoud F, Ukena C, Fries P, Böhm M (2013). Diagnostik und Therapie der Myokarditis. Der Kardiologe.

[4] Kristen AV, Dengler TJ, Katus HA (2007). Suspected cardiac amyloidosis: endomyocardial biopsy remains the diagnostic gold-standard. Am J Hematol.

[5] Prochorec-Sobieszek M, Bilinska ZT, Grzybowski J, Michalak E, Jakubowska E, Sobieszczanska-Malek M, Deptuch T, Walczak E, Wagner T, Walski M (2005). Cardiac amyloidosis diagnosed by endomyocardial biopsy. Clinical, histopathological, immunohistochemical and ultrastructural studies. Kardiologia Polska.

[6] Cooper LT, Baughman KL, Feldman AM, Frustaci A, Jessup M, Kuhl U, Levine GN, Narula J, Starling RC, Towbin J (2007). The role of endomyocardial biopsy in the management of cardiovascular disease: a scientific statement from the American Heart Association, the American College of Cardiology, and the European Society of Cardiology Endorsed by the Heart Failure Society of America and the Heart Failure Association of the European Society of Cardiology. Eur Heart J.

[7] http://www.ethikkommission.med.uni-goettingen.de/de/media/satzung-ethikkommission_2014-02.pdf. Accessed 12 Oct 2015.

[8] https://www.uni-luebeck.de/fileadmin/uzl_forschung/ethikkommission/Hilfreiches/Hilfe_WannVotumEinholen.pdf. Accessed 12 Oct 2015.

[9] Klingel K, Sauter M, Bock CT, Szalay G, Schnorr JJ, Kandolf R (2004). Molecular pathology of inflammatory cardiomyopathy. Med Microbiol Immunol.

[10] Aretz HT, Billingham ME, Edwards WD, Factor SM, Fallon JT, Fenoglio JJ, Olsen EG, Schoen FJ (1987). Myocarditis. A histopathologic definition and classification. Am J Cardiovasc Pathol.

[11] Klingel K, Stephan S, Sauter M, Zell R, McManus BM, Bultmann B, Kandolf R (1996). Pathogenesis of murine enterovirus myocarditis: virus dissemination and immune cell targets. J Virol.

[12] Mahrholdt H, Goedecke C, Wagner A, Meinhardt G, Athanasiadis A, Vogelsberg H, Fritz P, Klingel K, Kandolf R, Sechtem U (2004). Cardiovascular magnetic resonance assessment of human myocarditis: a comparison to histology and molecular pathology. Circulation.

[13] Feldman AM, McNamara D (2000). Myocarditis. N Engl J Med.

[14] Mason JW (2003). Myocarditis and dilated cardiomyopathy: an inflammatory link. Cardiovasc Res.

[15] D’Ambrosio A, Patti G, Manzoli A, Sinagra G, Di Lenarda A, Silvestri F, Di Sciascio G (2001). The fate of acute myocarditis between spontaneous improvement and evolution to dilated cardiomyopathy: a review. Heart.

[16] Maron BJ, Towbin JA, Thiene G, Antzelevitch C, Corrado D, Arnett D, Moss AJ, Seidman CE, Young JB, American Heart A (2006). Contemporary definitions and classification of the cardiomyopathies: an American Heart Association Scientific Statement from the Council on Clinical Cardiology, Heart Failure and Transplantation Committee; Quality of Care and Outcomes Research and Functional Genomics and Translational Biology Interdisciplinary Working Groups; and Council on Epidemiology and Prevention. Circulation.

[17] Kindermann I, Kindermann M, Kandolf R, Klingel K, Bultmann B, Muller T, Lindinger A, Bohm M (2008). Predictors of outcome in patients with suspected myocarditis. Circulation.

[18] Schwab J, Pauschinger M (2014). Myocarditis. Aktuel Kardiol.

[19] Caforio AL, Pankuweit S, Arbustini E, Basso C, Gimeno-Blanes J, Felix SB, Fu M, Helio T, Heymans S, Jahns R (2013). Current state of knowledge on aetiology, diagnosis, management, and therapy of myocarditis: a position statement of the European Society of Cardiology Working Group on Myocardial and Pericardial Diseases. Eur Heart J.

[20] Kuehl U, Schultheiss HP (2012). Myocarditis: early biopsy allows for tailored regenerative treatment. Deutsches Arzteblatt Int.

[21] Schönland SO (2006). Fortschritte in der Diagnostik und Therapie der Amyloidosen. Deutsches Ärzteblatt.

[22] Lim HE, Pak HN, Kim YH (2006). Acute myocarditis associated with cardiac amyloidosis manifesting as transient complete atrioventricular block and slow ventricular tachycardia. Int J Cardiol.

